# Health care workers’ experiences with implementation of “screen and treat” for cervical cancer prevention in Malawi: A qualitative study

**DOI:** 10.1186/s43058-020-00097-3

**Published:** 2020-12-14

**Authors:** Corrina Moucheraud, Paul Kawale, Savel Kafwafwa, Roshan Bastani, Risa M. Hoffman

**Affiliations:** 1 University of California Fielding School of Public Health, Los Angeles, CA USA; 2 African Institute for Development Policy, Lilongwe, Malawi; 3 Partners in Hope Medical Center, Lilongwe, Malawi; 4 University of California Geffen School of Medicine, Los Angeles, CA USA

**Keywords:** Cervical cancer, Screening, Global health, CFIR

## Abstract

**Background:**

Cervical cancer remains a major cause of mortality and morbidity in low- and middle-income countries, despite the availability of effective prevention approaches. “Screen and treat” (a single-visit strategy to identify and remove abnormal cervical cells) is the recommended secondary prevention approach in low-resource settings, but there has been relatively scarce robust implementation science evidence on barriers and facilitators to providing “screen and treat” from the provider perspective, or about thermocoagulation as a lesion removal technique.

**Methods:**

Informed by the Consolidated Framework for Implementation Research (CFIR), we conducted interviews with ten experienced “screen and treat” providers in Malawi. We asked questions based on the CFIR Guide, used the CFIR Guide codebook for a descriptive analysis in NVivo, and added recommended modifications for studies in low-income settings.

**Results:**

Seven CFIR constructs were identified as positively influencing implementation, and six as negatively influencing implementation. The two strong positive influences were the relative advantage of thermocoagulation versus cryotherapy (*Innovation Characteristics*) and respondents’ knowledge and beliefs about providing “screen and treat” (*Individual Characteristics*). The two strong negative influences were the availability of ongoing refresher trainings to stay up-to-date on skills (*Inner Setting*, *Implementation Climate*) and insufficient resources (staffing, infrastructure, supplies) to provide “screen and treat” to all women who need it (*Inner Setting*, *Readiness for Implementation*). Weak positive factors included perceived scalability and access to knowledge/information, as well as compatibility, leadership engagement, and team characteristics, but these latter three were mixed in valence. Weak negative influences were structural characteristics and donor priorities; and mixed but weakly negative influences were relative priority and engaging clients. Cross-cutting themes included the importance of broad buy-in (including different cadres of health workers and leadership at the facility and in the government) and the opportunities and challenges of offering integrated care (screening plus other services).

**Conclusions:**

Although “screen and treat” is viewed as effective and important, many implementation barriers remain. Our findings suggest that implementation strategies will need to be multi-level, include a diverse set of stakeholders, and explicitly address both screening and treatment.

**Supplementary Information:**

The online version contains supplementary material available at 10.1186/s43058-020-00097-3.

Contributions to the literature

- Despite the recommendation for “screen and treat” for cervical cancer prevention in low-resource settings—and the emergence of thermocoagulation as a treatment option—there have been few theoretically grounded qualitative studies on its implementation in high-burden countries.
- Providers in Malawi report considerable benefits of thermocoagulation versus cryotherapy, and enthusiasm for providing “screen and treat” -- but many challenges persist especially related to the Inner Setting, Process, and Systems.
- Care integration emerged as both a positive and negative factor, suggesting more work is needed to develop and evaluate implementation strategies that incorporate integration, particularly in high-burden contexts with resource-constrained health systems.

## Background

Routine screening is effective for reducing cervical cancer mortality [1–5], but in low-income countries, screening coverage remains low and cervical cancer burden is high [2, 5, 6]. In lower-resource settings like Malawi—which has the greatest cervical cancer burden in the world [6, 7]—the World Health Organization recommends a single-visit “screen and treat” (S&T) strategy using visual inspection with acetic acid (VIA) to examine the cervix and immediate removal of any abnormal cells (with referral to specialist care for more complicated cases) [3]. Many S&T programs use cryotherapy for lesion removal (an applicator freezes abnormal cells using low-temperature gas), although thermocoagulation (also called thermal ablation or cold coagulation, which uses a heated metal probe to destroy abnormal cells) is emerging as a safe, acceptable, and effective alternative approach [8–17]. Malawi clinical guidelines recommend S&T for all adult women [18, 19]—but very few Malawian women have ever been screened and fewer than half of screen-positive cases are treated [18, 20].

Evidence strongly supports the effectiveness and cost-effectiveness of S&T [4, 21, 22]. Although S&T was designed for resource-constrained systems, there are numerous implementation challenges, e.g., with cryotherapy supplies, human resources, and physical infrastructure, as well as patient-side barriers including awareness and affordability [23–31]. Previous studies about women’s experiences with S&T have identified challenges including supply/equipment stock-outs, lack of available providers, hesitations about male providers, and poor patient-provider communication [32–35]—but very little research has focused on the experiences of health care workers as providers of S&T.

This qualitative study examines providers’ experiences with the implementation of S&T using thermocoagulation in Malawi and is informed by the Consolidated Framework for Implementation Research (CFIR). The CFIR is a meta-framework that synthesizes constructs theorized to affect the implementation of interventions in the health care delivery context [36], and has been used to explore providers’ experiences with HPV vaccine implementation in Mozambique [37] and client experiences with cytology-based cervical cancer screening in the Dominican Republic [38] and following HPV DNA testing in Kenya [39]—but never to investigate the implementation of S&T nor thermocoagulation. This is a noteworthy gap in the literature, given the predominance of S&T in lower-resource settings and the recent adoption of thermocoagulation as a treatment modality.

## Methods

### Study setting

Malawi is a country in south-eastern Africa, with a population of approximately 18 million people [40]. Malawi has the highest cervical cancer burden in the world, with an age-standardized incidence rate of 72.9 cases per 100,000 women and an attributable mortality rate of 54.5 deaths per 100,000 women [6, 7]. Although screening uptake data in Malawi are scarce, both household surveys and facility-based data estimate that only approximately 20% of women have ever been screened [41, 42], and less than 40% of women who are eligible for lesion removal receive this care [18]. S&T services are offered at all levels of the Malawi health system—primary, secondary, and tertiary—and women with larger lesions or suspected of cancer are referred to one of four central (tertiary) hospitals [18]. Per the Malawi Ministry of Health guidelines, S&T can be performed by nurses, clinical officers, and medical doctors [43]. There is a national training course on S&T that includes both didactic and practical skills building, is oriented toward nurses and clinical officers, and is recommended to be administered over a 2-week period and later supplemented with mentorship and supportive supervision [43].

### Theoretical framework

The design of this qualitative study was informed by the CFIR. The CFIR posits that implementation may be influenced by factors related to the outer or inner setting, the characteristics of the intervention or of individuals, and the process of implementation [36]. Additionally, Means et al. recommended adding a sixth domain—characteristics of systems—when CFIR is applied in lower-resource contexts [44]. The CFIR was selected for this research because it comprehensively includes a broad range of factors potentially associated with implementation (both facilitators and barriers), which makes it well-suited for a topic lacking a robust preexisting literature that might indicate likely domains of interest.

### Site and participant selection

Interviews were conducted at three health facilities (two district hospitals and one mission hospital) in central Malawi that have been offering S&T with thermocoagulation since at least 2015, which is when a USAID-supported program to deliver cervical cancer screening services enabled consistent provision of services in these locations through support for supplies and equipment, and training and mentoring of providers for VIA and thermocoagulation. In 2015, there were an estimated 32 facilities in Malawi providing S&T [18]. The sites for this study were chosen from among those participating in the aforementioned USAID program. We focused on central Malawi for geographic feasibility of data collection and selected three sites that were representative of those implementing S&T in Malawi (1 large mission hospital, 1 large district hospital, and 1 smaller district hospital).

All health care providers with any experience administering VIA and thermocoagulation at these sites were invited to participate in an interview. No prospective participants declined to participate; thus, this sample represents the universe of available and eligible providers at the participating sites.

### Data collection

The interview guide was informed by the CFIR and tools available at www.cfirguide.org, plus modifications to reflect the low-resource environment of our participating sites [45] (see interview guide in Additional file 1). The guide included questions to touch on all domains of the CFIR. All interviews were conducted in Chichewa (the local language) by trained and experienced research assistants who were not affiliated with S&T programs or care delivery in order to minimize bias (1 male and 2 female). The interviews were audio recorded.

### Data analysis

Highly experienced research staff transcribed and translated the audio-recorded interviews; these transcripts were coded (by CM) using an a priori codebook developed from the CFIR Guide codebook [46] plus modifications recommended for low-resource settings [44]. NVivo software was used (QSR International, v11). We used content analysis methods and a deductive approach to evaluate the transcripts for all CFIR constructs; notes and quotations from the interviews were used to create a memo corresponding to each CFIR construct identified, and these memos were analyzed to assess valence (positive or negative influence or mixed, i.e., disagreement about valence across respondents) and strength (strong or weak influence) of the construct.

### Ethical review

All participants gave oral informed consent to participate and for audio recording. This study was approved by the Institutional Review Board at the University of California Los Angeles and the Malawi National Health Sciences Research Committee.

## Results

Ten interviews were conducted with S&T providers (Table 1); most were nurses (*n* = 9) and female (*n* = 7) and had been providing S&T since at least 2015 (*n* = 7). Respondents were asked about cervical cancer screening programs at their facility, and all spoke specifically about S&T using thermocoagulation as the primary approach they use. Most respondents (*n* = 7) had experience also using cryotherapy, either previously or currently as an alternative method.

**Table 1:** Respondent characteristics (*n* = 10)

Role	
Nurse, community health nurse, or nurse/midwife	9
Clinical officer	1
Experience performing “screen and treat”	
< 1 year	2
1–2 years	1
3–4 years	6
5+ years	1
Sex	
Female	7
Male	3

Based on the transcribed interviews, seven CFIR constructs were identified as positively influencing implementation and six as negatively influencing implementation (Table 2). This section details findings by construct rated for valence and strength. Definitions of all included CFIR domains and constructs can be found in Additional file 1.

**Table 2:**
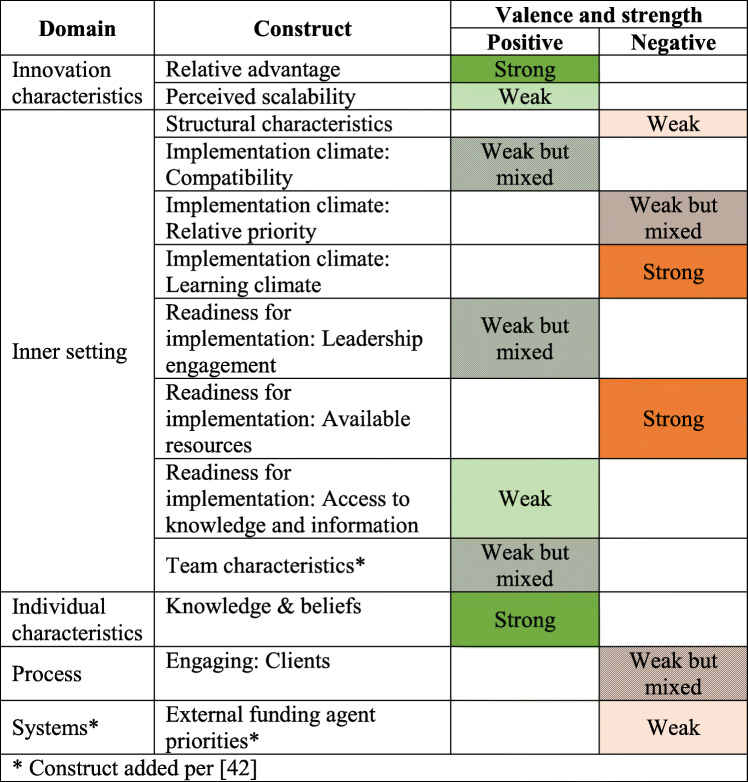
CFIR domains and constructs identified in this analysis

* Construct added per [41] ; Green = positive valence; Orange = negative valence

### Innovation characteristics

#### Relative advantage (strong positive factor)

Overall, health care providers were enthusiastic about S&T and the benefits of same-day lesion removal. This was mentioned by respondents at all sites and both by experienced and newer S&T providers.

VIA is a right-away process. We do treatment right there while chatting with the woman, while they are awake… They are able to walk back home without any problems. (Nurse, female, 8 years’ experience with S&T)

Those respondents who were experienced with different lesion removal techniques saw thermocoagulation as preferable to cryotherapy. This was largely attributed to being less resource-intensive, easier to operate, and fast, which can lessen workload in very overburdened facilities.

You can use [the thermocoagulator] with a power bank so even if you don’t have lights, you can use it… Since we started using thermocoagulation, we help more women. (Nurse, female, 3 years’ experience with S&T)

The [thermocoagulation] machine is small and portable, so we can carry it and do campaigns… The cryotherapy cylinder is huge so carrying it around would be a big task. (Nurse, female, 8 years’ experience with S&T)

#### Perceived scalability (weak positive factor)

Respondents felt as though S&T should be scaled up. This included recommendations for more service days at implementing health facilities, more providers trained in S&T, and community-based campaigns that bring services closer to where women live (mobile screening or at lower-level health centers)—which were also seen as advantageous for increasing screening coverage and reducing burden at higher-level health facilities.

CC screening is not hard, it doesn’t need a lot of things. Health centers should be screening and if they find a problem they can treat it… [which would] reduce overcrowding here. (Nurse, male, 3 years’ experience with S&T)

### Inner setting

#### Implementation climate: learning climate (strong negative factor)

Nearly every respondent expressed disappointment and frustration that they had not received any formal refresher training since the first time they learned about S&T. Providers worried they were losing practical skills, falling behind the “cutting edge” of clinical knowledge and felt demotivated by the lack of additional training. This was most commonly mentioned by respondents who began providing S&T in 2015 (when the USAID-supported cervical cancer screening program began).

Sometimes you seem to be lagging behind, when you meet a fellow provider who is talking about something new. It is a problem that we never get sent to do a refresher course. [I just apply] what I learned a long time ago. (Nurse, female, 4 years’ experience with S&T)

#### Readiness for implementation: available resources (strong negative factor)

At every site, respondents discussed how shortages of S&T-trained health workers are affecting the availability and use of services. Some also spoke about how VIA providers are juggling many clinical duties, and may find it hard to focus on S&T.

The cervical cancer program here is not working how it is supposed to work. Last week we had only 1 or 2 providers… [Women] end up being disappointed and they don’t come back. (Nurse, female, 3 years’ experience with S&T)

The providers are few, and we also have other duties to do… Like for me I also work at the surgical theater, so when I am on [VIA] duty they locate me at the theater. To leave things there is difficult so people here [at VIA] will just wait and may be sent back home because it will be impossible for me to screen them. (Nurse, male, 3 years’ experience with S&T)

Other resource constraints included utilities such as electricity and water. This was more commonly mentioned by respondents at district hospitals.

Here in [Facility X] water supply is a challenge. When they shut down the water, it includes here at the hospital too. How will you provide VIA when the speculums are not cleaned, and when there is no water to wash your hands? (Nurse, female, 2 years’ experience with S&T)

We don’t have torches. We sometimes use a phone, but the brightness of the phone is not enough… These are small things that make the work so hard. (Nurse, female, 2 years’ experience with S&T)

Additionally, supply shortages were mentioned, particularly for speculums; this was also more common at the district hospitals than the mission hospital.

It’s very painful to tell someone that they should go home because equipment has run out, because they have been waiting since morning. (Nurse, male, 3 years’ experience with S&T)

We have few speculums and we send people back because we don’t have enough. Work is good when there is equipment to use. You enjoy the work when there are enough resources. But if you come to work and find there aren’t enough resources, you still work but you are unhappy and you lose interest. (Nurse midwife, female, less than 1-year experience with S&T)

#### Implementation climate: compatibility (weak positive but mixed factor)

It was common for respondents to discuss synergies with other services at their health facilities—however, integration can impose new challenges, such as difficulties in juggling additional responsibilities and in reconciling different levels of care complexity. Concerns about care integration were mentioned solely by respondents at the district hospitals.

Like the way HIV came to antenatal services, at first we were just testing women, but later we started dispensing antiretroviral therapy. It’s the same way VIA has come in. We have added to our work load. (Nurse, female, 2 years’ experience with S&T)

#### Readiness for implementation: leadership engagement (weak positive but mixed factor)

Most respondents felt as though facility leadership had been engaged in promoting S&T activities—but some felt as though a lack of leadership was affecting the implementation of S&T. This was mentioned by both more and less experienced S&T providers, and across all sites.

This is why we have a cervical cancer clinic – because the leadership saw the importance of screening for all women that are in the reproductive age… The leadership has a great vision in the VIA clinic. (Nurse, female, 8 years’ experience with S&T)

As providers, we do not even know the national cervical cancer coordinator… The Ministry [of Health] should hear us when we say that cervical cancer is real but the capacity is low, which is impeding the progress of the program. (Nurse midwife, female, 3 years’ experience with S&T)

#### Readiness for implementation: access to knowledge and information (weak positive factor)

VIA providers discussed asking each other questions first and then escalating their queries if needed, including using WhatsApp and other messaging services.

#### Team characteristics (weak positive but mixed factor)

A few respondents mentioned teamwork as a facilitator of S&T implementation—but one respondent spoke strongly about an apparent lack of teamwork among higher-cadre providers at her facility.

After people are screened and we think they have cancer, we send them to the doctors, our bosses. Some patients come back to say they were not assisted… It shows that some of the clinicians are not sure about the way forward, like how to harvest cells for a biopsy. We are referring the clients to the doctors… [but] they do not have the knowledge or it may be negligence, I don’t know. (Nurse midwife, female, 3 years’ experience with S&T)

#### Structural characteristics (weak negative factor)

Some facilities have S&T-specific rooms, and others share space with other programs. Respondents noted challenges with insufficient space for S&T and lack of privacy.

We only have one door, so women are waiting outside that door. If you need to call someone to help you, women at the door might get suspicious that something might be wrong inside. There is no privacy. (Nurse, female, 4 years’ experience with S&T)

Two rooms were meant for VIA but because we have so many programs, we do use it for other things also. (Nurse, female, 2 years’ experience with S&T)

#### Implementation climate: relative priority (weak negative but mixed factor)

Some respondents said they felt that S&T is a priority for their organization; reasons for this included national- or district-level commitment, synergies with HIV, and disease burden. However, as noted across other constructs, several respondents felt as though S&T is not a priority. This was mentioned by at least one respondent at each facility.

Health service providers forget to tell them about cervical cancer. I feel that we don’t prioritize VIA services at this hospital… I think the management doesn’t really take cervical cancer screening seriously. (Nurse, female, 4 years’ experience with S&T)

### Individual characteristics

#### Knowledge and beliefs (strong positive factor)

Most respondents said that they felt encouraged by the tangible impact of S&T, particularly owing to immediate treatment, and spoke passionately about their satisfaction with this aspect of their work. This was mentioned by veteran and novice S&T providers, and across sites.

When we screen them and find that one is VIA positive I feel happy because I know that we have saved the life of that woman. She is the one taking care of the home, looking after the children and some are working – so when we diagnose them we have given them another chance at life. (Nurse, female, 8 years’ experience with S&T)

I enjoy this work and it makes me happy, because I see that we are able to protect a woman from an illness that may kill her if we do not discover it faster. (Clinical officer, male, less than 1 year experience with S&T)

Providers also felt as though training had well-prepared them for implementing S&T although (as noted above, *learning climate*) the lack of refresher trainings was cited as a concern.

### Process

#### Engaging: clients (weak negative but mixed factor)

Several respondents felt that more should be done to increase knowledge about S&T (including outside the health facility setting), especially to address common concerns about screening being painful or dangerous and lack of support from husbands.

Some say they fear their husbands. When we insert the speculum, some women say ‘the vagina wall will enlarge so my husband will think I had sex with another man. He won’t believe that I went to the hospital and you inserted a speculum.’ (Nurse, female, 4 years’ experience with S&T)

Some providers noted that fears like these had dissipated in recent years and that women are now sharing information about their experience with other women, which dispels rumors and increases the demand for S&T.

After people are screened, they go back to the village and tell their friends and family about the advantage of screening… The same clients that we are helping are the ones who are also helping us spread the message in the villages. (Nurse midwife, female, less than 1 year experience with S&T)

### Systems

#### External funding agent priorities (weak negative factor)

Although mentioned by only a couple of respondents, both felt as though funding for cervical cancer prevention is insufficient in Malawi—and that this reflects a relatively low priority among donors.

[This] is a program that is left behind. It isn’t supported or funded. There isn’t interest. But it’s a program that is very helpful and can save a lot of people. (Nurse, male, 3 years’ experience with S&T)

## Discussion

Through interviews with health care providers responsible for providing cervical cancer screening services in Malawi, this study identified implementation barriers and facilitators that resonate with themes from the broader literature, including insufficient human resources [23–25, 27–29] and lack of space and physical infrastructure [25, 27, 29]. Human resource challenges were reported across all facilities; however, supply and infrastructure challenges were more commonly mentioned at public sector facilities (district hospitals) rather than private sector (mission) hospitals.

Health care workers are valuable key informants both because they are highly informed about implementation realities and because they may be influential toward the ultimate successes or failures of implementation. Although health care providers in this study noted a number of advantages to using thermocoagulation in “screen and treat” (S&T) programs—including being easier and faster to use than other methods—they nonetheless reported many of the previously identified challenges of implementing S&T using cryotherapy, suggesting that, in a low-resource setting, introducing an improved technology may have limited impact without accompanying health system strengthening efforts. We also identified new implementation challenges related to S&T, including the lack of refresher trainings. As new programs scale up, ensuring continued training opportunities—to practice skills and learn new clinical advances—should be a budgetary and programmatic priority.

This study also lends supportive evidence to proposed new domains for CFIR in lower-resource contexts, such as donor priorities. To our knowledge, only one other study has cited donor involvement, and the subsequent competing priorities faced by Ministries of Health, as a barrier to implementation of S&T [24]. This study did not interview policymakers or other stakeholders directly involved in donor relations, so these findings represent impressions from key informants without first-hand knowledge of discussions about donor priorities—however, such perceptions may affect attitudes toward implementation and should nonetheless be considered. Additionally, our results suggest that cooperation and engagement of providers across cadres is important; this has been infrequently identified in the S&T literature but merits further exploration and possible intervention. Given the high turnover of health workers, successful S&T implementation may require stronger systems of communication, referral, and feedback to ensure continuity of care.

Studies from sub-Saharan Africa have recommended integrating cervical cancer screening into other health services, particularly HIV care [26, 47–49]. But our analysis, alongside findings from other studies of cervical cancer integration [50, 51], suggests that smooth and successful integration will require adequate funding, robust monitoring and referral systems, a sufficient pool of well-trained and available health workers (across cadres, to enable task shifting), and ongoing demand from clients [51–55]. There may also be hidden “costs” of integration, for example increasing stigma if other services are bundled with HIV treatment [53, 54], and providers may need special training in the process of integration (not only each stand-alone service, but the provision of integrated care) as well as appropriate support and accountability for offering comprehensive services [54]. Concerns about integration were most prevalent among respondents at public sector facilities; public sector facilities in Malawi have scarcer human and other resources than the mission hospitals (private sector facilities), so clinicians at these sites may find it particularly challenging to accommodate new tasks [56].

We add new evidence to a relatively small literature using implementation science to study and improve care for cervical cancer prevention in sub-Saharan Africa [51, 57, 58]. Some limitations should nonetheless be noted. First, data were collected from sites experienced with S&T, so factors unique to the start-up phase are not reflected here. As these sites were selected from among those providing services in central Malawi, there may also be geographic or other intra-national differences that we were unable to explore in this analysis. Similarly, most respondents were highly experienced with S&T; future work should therefore explore whether the duration of experience with S&T affects perceived implementation facilitators and challenges. Two respondents were new to providing S&T (during the last year) but as we did not ask specifics about when they began, it is possible they were not sufficiently experienced to offer comparable insights to the more seasoned providers. Additionally, respondents were interviewed at their place of work so may have under-reported implementation challenges; we tried to mitigate such reporting bias by using highly skilled qualitative interviewers who are experienced with collecting data from health professionals. A final limitation to note is that this analysis focused solely on the perspective of health care workers—who are immersed in implementation so provide valuable insights and are essential for implementation success—but future research should incorporate multiple perspectives, such as facility managers and leadership, key policy decision-makers, and clients.

## Conclusions

Cervical cancer prevention is a global priority [59, 60]. We have a toolkit of evidence-based interventions to address this inequity—now we must develop robust implementation strategies to reach the women most in need [61, 62]. This study highlights facilitators and barriers amenable to change, such as ensuring adequate infrastructure, supplies, and support for health care providers; carefully designing integrated care strategies; and encouraging buy-in from a variety of stakeholders including other clinicians and leadership at the facility, subnational and national levels.

## Supplementary Information

**Additional file 1: Appendix 1.** Interview questions. **Appendix 2.** Definitions of all constructs used in this analysis

## Data Availability

The datasets generated during the current study are not publicly available in order to protect respondent confidentiality, but are available from the corresponding author on reasonable request.

## Abbreviations

CFIR: Consolidated framework for implementation research
S&T: Screen and treat
USAID: United States agency for international development
VIA: Visual inspection with acetic acid
