# Supplementary material for: Health care workers’ experiences with implementation of “screen and treat” for cervical cancer prevention in Malawi: A qualitative study
Source: Implement Sci Commun. 2020 Dec 14;1:112. doi: 10.1186/s43058-020-00097-3 (PMC7734769; doi:10.1186/s43058-020-00097-3)
Supplement: Supplementary file 1 — Additional file 1: Appendix 1. Interview questions. Appendix 2. Definitions of all constructs used in this analysis [file 43058_2020_97_MOESM1_ESM.docx]

Appendix 1: Interview questions

1. What is your clinical title/role?
2. For how many years have you been conducting cervical cancer screening?
3. What methods does this health facility use for cervical lesion removal (prompt if needed: thermocoagulation, cryotherapy, colposcopy, etc.)? Have you used any other methods in your work?
   1. If provider has experience using more than one method: What do you feel the advantages are of [method A] versus [method B]? What are the relative challenges? Is there one you think is better than the others? If yes, what makes it better?
4. How do you think the CC program is going at this facility?
   1. Do you think it is changing people’s health? Why?
5. How are patients receiving the CC program? What problems do you think patients are facing in participating in the CC program?
6. How are staff at this hospital supporting implementation of CC screening?
7. How is CC screening integrated into current processes? Are other health services negatively or positive affected by the CC screening program?
8. For leadership at this facility, how does CC screening compare to other programs in relation to importance and value (prompt if needed: resources, attention, training, etc.)?
9. For you as staff at this hospital, how do you feel about CC screening in your daily duties? How will you juggle competing priorities in your own work?
10. How does infrastructure of this facility affect the implementation of CC screening? (prompt if needed: organization, hospital size, staff, physical layout of the hospital) How do you work around these structural challenges?
11. What changes do you think would help make the CC program most effective in your setting? In your opinion, do you think these changes are possible? Why?
12. Are there components that should not be altered? If so, which and why?
13. What kinds of incentives are there to help ensure that CC screening implementation is successful?
14. Have you/your unit/your organization set goals related to the implementation of the intervention? [If yes] What are the goals?
15. Do you have sufficient resources to implement and administer CC screening (prompt if needed: money, supplies, space, staff, time, etc.)? Why or why not (please describe)?
16. What kind of CC-related training have you received? Do you feel the training prepared you to carry out the roles and responsibilities expected of you? Please explain.
17. Are there any continuing education activities related to cervical cancer (CPDs) that are done? If so, how often? Have you participated?
18. Who do you ask if you have questions about the CC program or its implementation? How available are these individuals?

Thank you very much for agreeing to speak with me today. Do you have any additions or questions, before we conclude?

Appendix 2: Definitions of all constructs used in this analysis

| Domain | Construct | Definition |
| --- | --- | --- |
| Innovation characteristics | Relative advantage | Perception of the advantage of implementing S&Thermocoagulation versus an alternative screening approach, or S&T using cryotherapy |
|  | Perceived scalability* | How/how much could S&Thermocoagulation implementation be expanded to reach different groups (geographies, service settings) |
| Inner setting | Structural characteristics | Organization’s structure, architecture, age, maturity, size |
|  | Implementation climate: Compatibility | How does S&Thermocoagulation (meaning and values) align with individuals’ own norms, values, and perceived risks and needs; and fit with existing workflows and systems |
|  | Implementation climate: Relative priority | Do stakeholders agree about the importance of S&Thermocoagulation within the organization |
|  | Implementation climate: Learning climate | How/how much do leaders and team members seek, obtain and use new approaches, knowledge, reflection and evaluation |
|  | Readiness for implementation: Leadership engagement | Are leaders and managers committed and involved in implementation of S&Thermocoagulation |
|  | Readiness for implementation: Available resources | Are resources dedicated to implementation of S&Thermocoagulation |
|  | Readiness for implementation: Access to knowledge and information | Is information about S&Thermocoagulation readily available and accessible, and can it be incorporated into work tasks |
|  | Team characteristics* | Features of a team (who is on the team, their traits, and processes) |
| Individual characteristics | Knowledge & beliefs | One’s opinions about S&Thermocoagulation, and factual knowledge about it |
| Process | Engaging: Clients | Approaches to involve clients in the implementation of S&Thermocoagulation |
| Systems* | External funding agent priorities* | How/how much do donor preferences/agendas influence implementation of S&Thermocoagulation |

Definitions adapted from those in the CFIR Codebook, and from Means et al 2020 (noted with *)

S&Thermocoagulation: Screen & Treat using thermocoagulation
